# A corpus of CO_2_ electrocatalytic reduction process extracted from the scientific literature

**DOI:** 10.1038/s41597-023-02089-z

**Published:** 2023-03-29

**Authors:** Ludi Wang, Yang Gao, Xueqing Chen, Wenjuan Cui, Yuanchun Zhou, Xinying Luo, Shuaishuai Xu, Yi Du, Bin Wang

**Affiliations:** 1 Laboratory of Big Data Knowledge, Computer Network Information Center, Chinese Academy of Sciences, Beijing, 100083 China; 2 CAS Key Laboratory of Nanosystem and Hierarchical Fabrication, National Center for Nanoscience and Technology (NCNST), Beijing, 100190 China; 3 University of Chinese Academy of Sciences, Beijing, 100049 China

**Keywords:** Electrocatalysis, Scientific data

## Abstract

The electrocatalytic CO_2_ reduction process has gained enormous attention for both environmental protection and chemicals production. Thereinto, the design of new electrocatalysts with high activity and selectivity can draw inspiration from the abundant scientific literature. An annotated and verified corpus made from massive literature can assist the development of natural language processing (NLP) models, which can offer insight to help guide the understanding of these underlying mechanisms. To facilitate data mining in this direction, we present a benchmark corpus of 6,086 records manually extracted from 835 electrocatalytic publications, along with an extended corpus with 145,179 records in this article. In this corpus, nine types of knowledge such as material, regulation method, product, faradaic efficiency, cell setup, electrolyte, synthesis method, current density, and voltage are provided by either annotating or extracting. Machine learning algorithms can be applied to the corpus to help scientists find new and effective electrocatalysts. Furthermore, researchers familiar with NLP can use this corpus to design domain-specific named entity recognition (NER) models.

## Background & Summary

Electrocatalysis has garnered much attention in reducing fossil fuel consumption, decreasing greenhouse gas emissions, and producing sustainable fuels and chemicals^1,2^. Critical to realizing these goals is the development of improved electrocatalysts with high activity and selectivity for the target product. In general, the property of catalysts depends on their compositions, structures, and regulation methods^3,4^, and thus there is enormous synthesis and regulation space for catalyst exploration. Although extensive efforts have been devoted to the design and development of novel electrocatalysts^5,6^, most of the previous exploration is based on heuristics and experience and still lacks effective design guidelines. Furthermore, it seems unreasonable to conduct enough attempts to cover a majority of the synthesis and regulation space to explore novel catalysts, even with the aid of high-throughput synthesis techniques.

The establishment of realm-specific datasets is a crucial step to promote the development of catalysts. A few existing catalyst datasets are built from density functional theory (DFT) calculations and mainly encompass features related to surface adsorption and electronic structure^7,8^. Researchers in catalytic science have proposed various kinds of descriptors for catalyst screening through a mass of calculations^9–11^. However, the real surface structure of catalysts is not ideal as theoretical calculations assume and is fairly complex, thus lowering the reliability of these datasets for catalyst design. In fact, an enormous amount of knowledge has been hidden in a large volume of scientific publications. If the concerned information related to catalysts can be extracted and collected into datasets, the efficiency of developing new catalysts can be greatly improved.

Compositions, structures, regulation methods, and properties that can describe specific catalysts generally exist in the unstructured and heterogeneous form of scientific literature. Data-driven approaches exhibit great potential to deal with these data. These approaches can complement experimental and theoretical studies and have been successfully applied in materials discovery^12–14^, material synthesis approaches^15,16^, and the interpretation of experimental spectra^17^. However, manual extraction of these data is nearly impractical and costs too much labor^18^. Natural language processing (NLP) and text-mining approaches have made great progress in the past decades, and various cutting-edge tools have been employed in biology, chemistry, and materials science^19–22^.

**Fig. 1:**
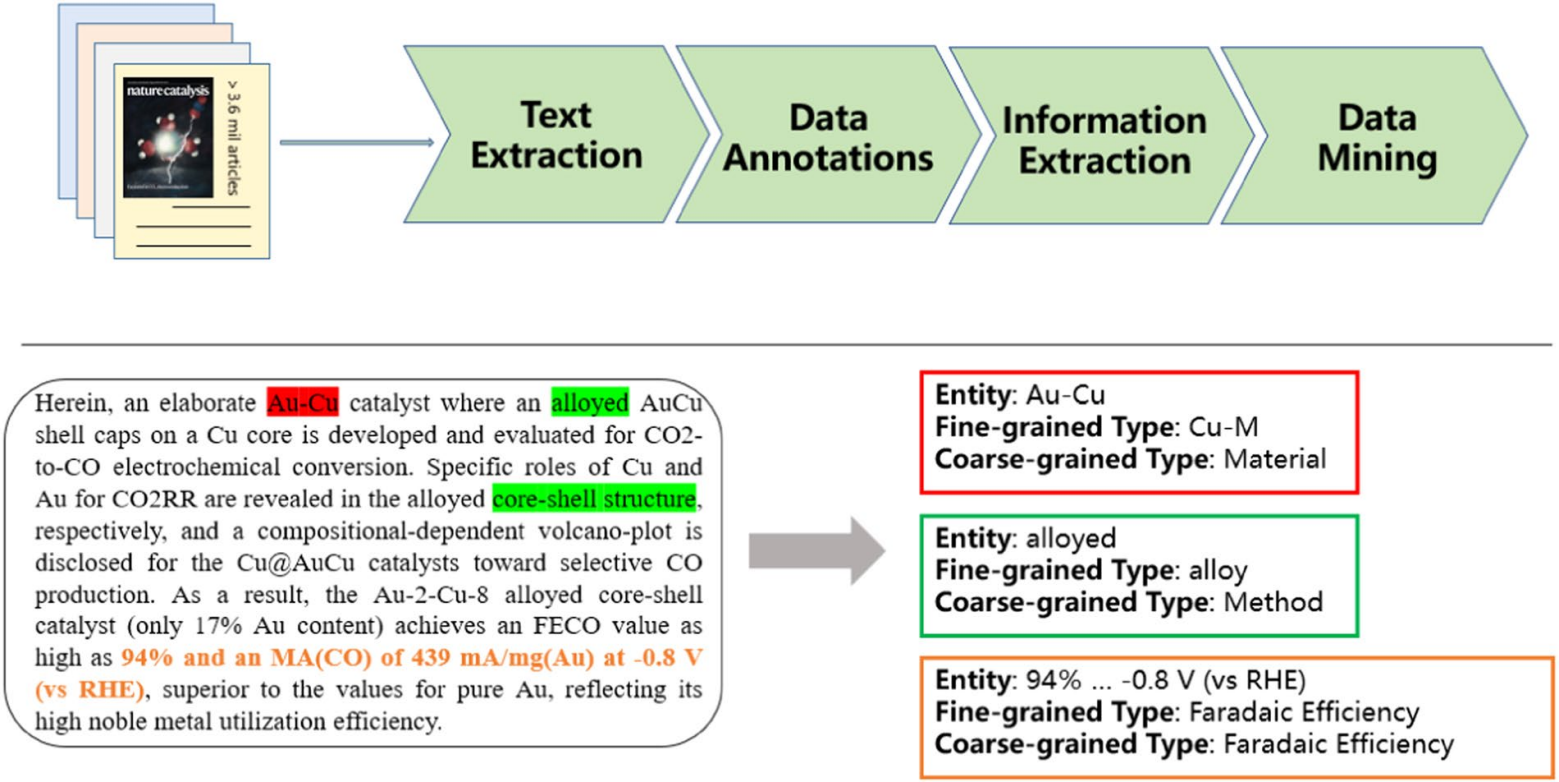
Extract pipes and samples. Top panel: Schematic diagram of standard text mining pipeline: (i) Collect papers by keyword search; (ii) Expert notes to build a benchmark corpus; (iii) Extract key information of the synthesis process and build an extended corpus; (iv) Stored in a database for future data mining. Bottom panel: Sample entities extracted from the summary.

In this data descriptor, we present an open-source corpus of electrocatalytic CO_2_ reduction. This database contains two types of corpus: (1) the benchmark corpus, which is a collection of 6,086 records extracted from 835 publications by catalysis postgraduates; (2) the extended corpus, which includes 145,179 records extracted from the full text of the 372 literature by intelligent model. In the benchmark corpus, we extracted nine types of knowledge, including material, regulation method, product, faradaic efficiency, cell setup, electrolyte, synthesis method, current density, and voltage. The extended corpus contains four types: material, regulation method, product, and faradaic efficiency. Moreover, the extended corpus was evaluated and revised by domain experts. A schematic of the pipeline devised for this extraction is shown in Fig. 1.

The advantage of the benchmark corpus is that it is a dataset annotated entirely by domain experts, thus the reliability and accuracy of its label can be guaranteed to a certain extent. Therefore, this kind of corpus can be used as a benchmark to guide the evaluation of NLP systems. The extended corpus, on the other hand, has the advantage of an automatic annotation system that can save the labor of manual annotation. Its extensive data resource can help experts to derive further information from it and provide guidance for some downstream tasks, such as faradaic efficiency prediction models.

## Methods

In the current work, we built a more advanced extraction pipeline (Fig. 1) that combines manual annotations and various advanced machine learning and NLP techniques to extract complete data for CO_2_ electrocatalytic reduction process from scientific literature. We first collected literature related to copper-based catalytic CO_2_ reduction procedures following a series of progressively finer-meshed filters. Then according to predefined entity labels, we published a manually annotated benchmark corpus and an automatically annotated extended corpus. The final resulting dataset can be used for domain data mining and further downstream NLP tasks. Each of the steps is described in detail below.

### Content acquisition

**Fig. 2:**
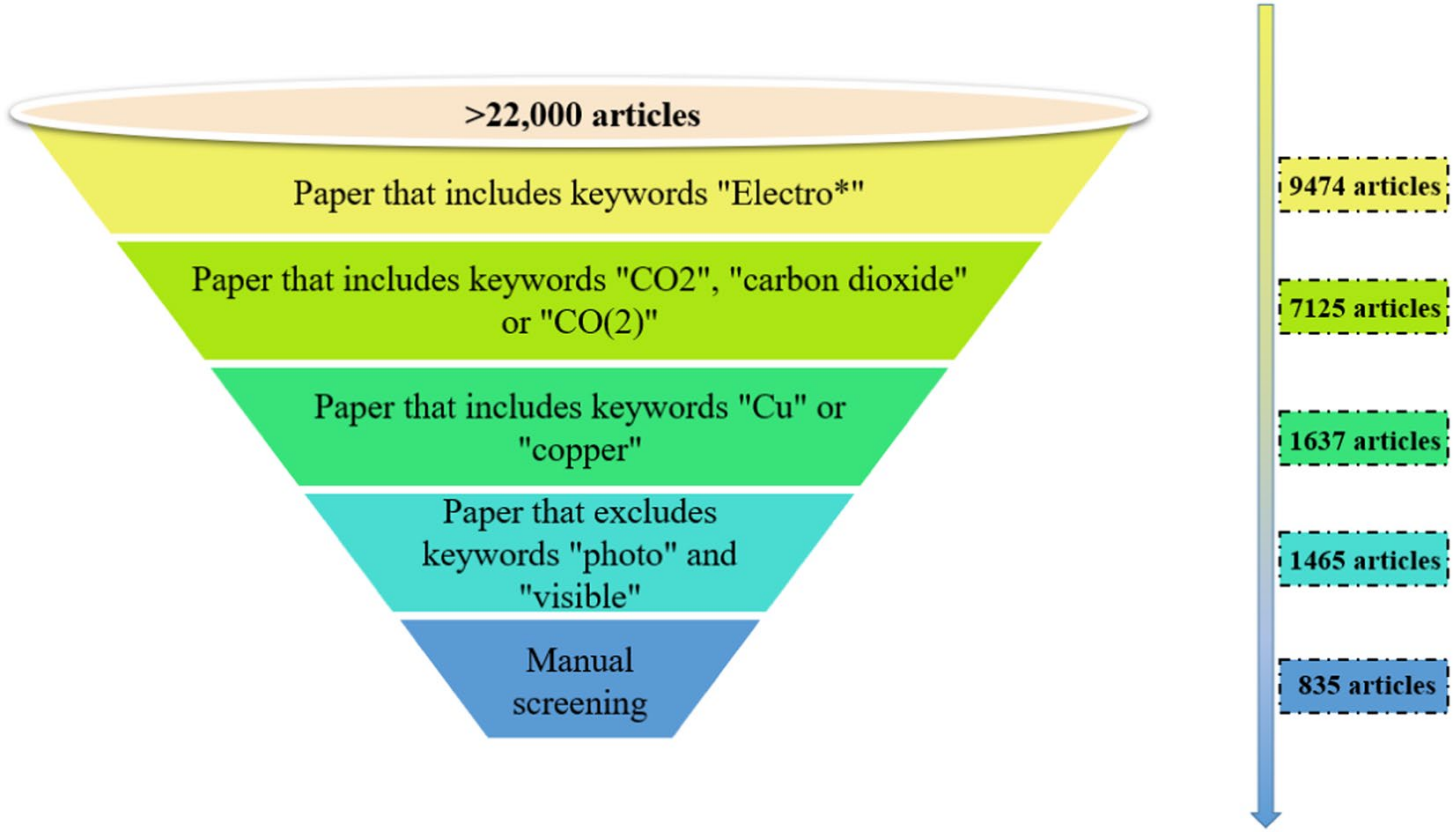
The process of literature screening.

The first step in the database generation workflow was using Web of Science to find the DOIs of scientific literature that will be used in the following steps. Specifically, over 22,000 metadata of articles were exported from Web of Science using the keywords “CO_2_”, “Reduction” and “Electro*” as subject index, such as article title, article DOI, article abstract, etc. Web of Science provides filtering and export functions on the website. The metadata of literature exported is then filtered step-by-step according to rules defined by experts, with each step of the filtering process consisting of a simple regular expression query^23^.The process of literature screening is illustrated in Fig. 2. The title of every article was queried for words starting with “electro”, followed by any number of characters or whitespace, which yielded 9,474 articles; The title of every article was queried for words “CO_2_”, “carbon dioxide” or “CO(2)”, which yielded 7125 articles; The title of every article was queried for words “Cu” or “copper”, which yielded 1637 articles; The title of every article was queried for words “photo” or “visible” and then removed, which yielded 1465 articles. Finally on this basis, combined with further manual screening by experts, 835 articles were obtained on experimental works related to the electrocatalytic reduction of CO_2_ over copper-based catalysts.

After the filtered step, the related 835 publications were downloaded manually from the web according to their DOI. These publications were obtained through agreements with publishers Elsevier, the Royal Society of Chemistry, American Chemical Society, Wiley, Acta Physico-Chimica Sinica & University Chemistry Editorial Office (Peking University), MDPI, the Electrochemical Society, Springer Nature, Informa, Hindawi Limited, Frontiers Media SA, China Science Publishing & Media Ltd., IOP publishing, NACE International, Proceedings of the National Academy of Sciences, Shanghai Institute of Ceramics, American Institute of Physics, American Scientific Publishers, the Chemical Society of Japan, the Electrochemical Society of Japan, Journal of New Materials for Electrochemical Systems, HARD Publishing Company, Taylor & Francis, American Association for the Advancement of Science (AAAS), ESG, Sycamore Global Publications, from which we received permissions to download the articles. For each publisher, we manually identified all materials science related journals available for download. We acquired papers in PDF format, which include the full text of the article as well as its metadata such as article title, public year, authors, etc. After filtered step described above, we imported the related articles to AutoDive, our self-developed annotation tool, which allows experts to annotate on PDF format directly.

### Full-text preparation

The full papers were operated differently according to the way the different corpora were constructed. As the annotation tool AutoDive is an online annotation platform, it is only necessary to import the literature into the platform in PDF format, organised in their DOI order, so that the experts can annotate entities directly. The extended corpus contains automatically generated entities based on the full text of the collected articles above. We used a PDF parsing tool, PyMuPDF library^24^, to automate the batch extraction processing of these literature data. Because the processed documents contained irrelevant markups, we developed a customized function for parsing article markup strings into text paragraphs while keeping the structure of paper and section headings.

### Entity annotation

The definition of regulation methods and related properties for the electrocatalytic reduction of CO_2_ is the key challenge in constructing the benchmark corpus. A prerequisite for the manual annotation for the provided corpus was that annotators had to have a background in CO_2_ electrocatalytic reduction to guarantee that the annotations are correct. Thus we invited 5 postgraduates with an average experience of at least 3 years in experimental catalysis from National Center for Nanoscience and Technology to do the work after the annotation tool training.

An easy-to-use annotation tool with graphical user interface which allows labeling of text efficiently and consistently is crucial and necessary. We found that on-site annotation in PDF format is an effective way after consulting domain experts. Thus, we explored alternative ways on how to present the documents to the annotator in a way that is supported by existing annotation tools. Finally we decided to adapt our own annotation tool, AutoDive, as the application for the construction of this corpus. AutoDive provides the label interface in the form of PDF, which can ensure the layout of the original documents that can keep the original habit of reading literature. This tool does not require local installation on the curators side and can be used through a web-browser to make the annotation process as easy and fast as possible.

**Fig. 3:**
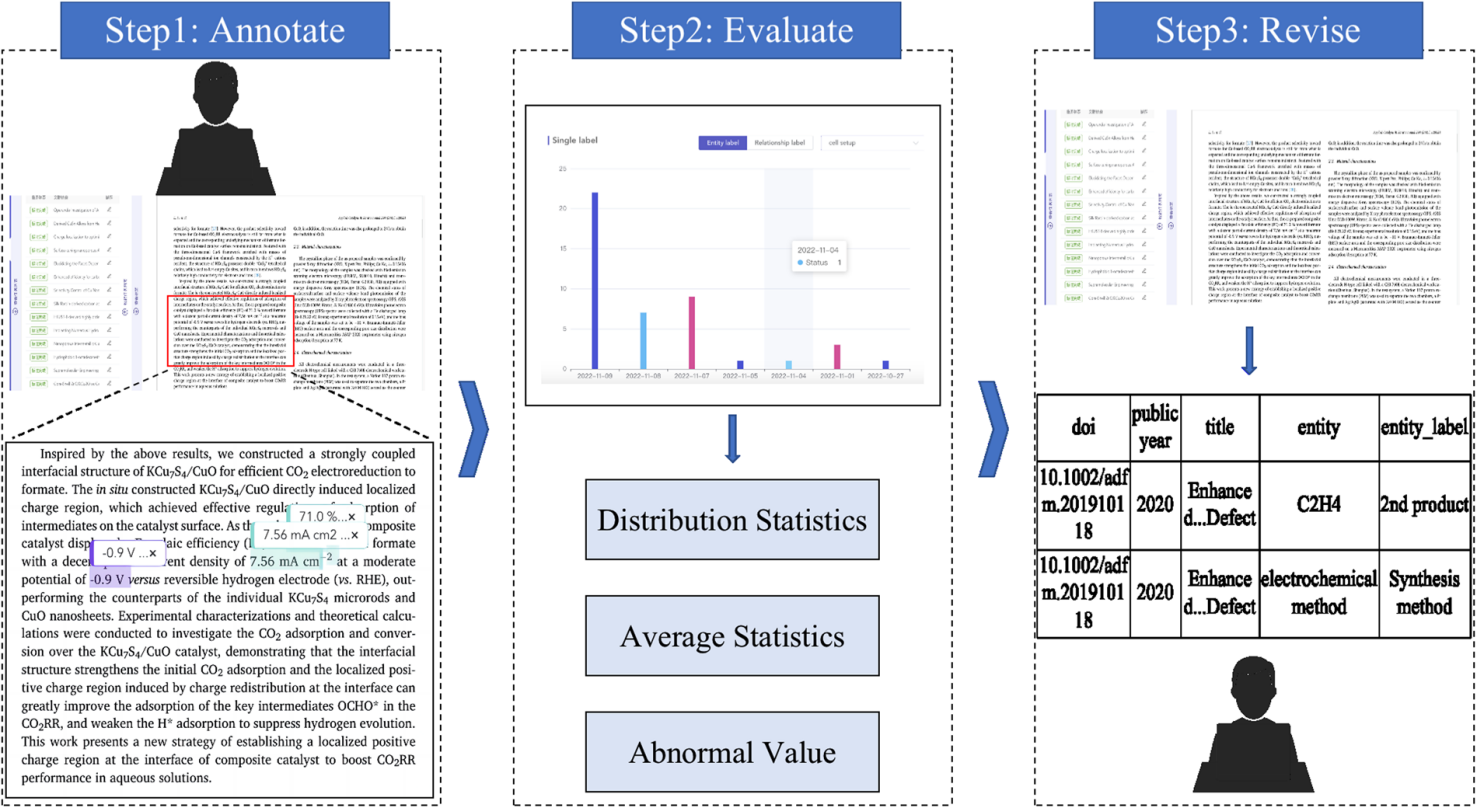
Overview of the construction of the benchmark corpus process.

Figure 3 provides a general flowchart of the annotation process. The main three steps of the annotation process are annotate, evaluate and revise:

**Stage 1: Annotate**. As mentioned before, we invited 5 annotators who have research background of electrocatalysis to annotate the entities with AutoDive followed by the guidelines. The documents are randomly and evenly assigned to these annotators by a senior expert. Three important things are emphasized in the annotation guidelines. The first is what kind of entity is needed to label. The second is the mention boundaries of those labels. The third is how to classify those mentions into label categories.

**Stage 2: Evaluate**. After manual annotation, we used multiple statistical methods to evaluate the quality of annotation results, such as distribution statistics, average statistics (for numerical value) and abnormal value statistics, etc. The evaluate results were provided to the senior expert for quality verification. The senior expert tagged the entity annotation which maybe incorrect.

**Stage 3: Revise**. The AutoDive tool can export the annotated data in CSV format, which is provided to annotators to revise and correct the mis-identified annotations and add missing entity label manually.

The annotation data underwent three rounds of modification in this project. Finally, we associated the all kinds of labeled entity and meta data of paper for further analysis, as well as to refine the annotation data.

### Entity extraction

**Fig. 4:**
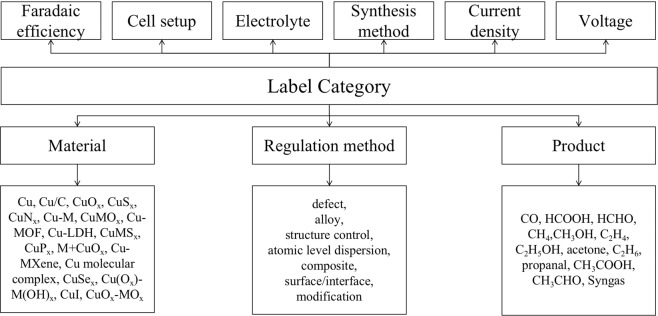
Nine kinds of label categories with three of them show specific subclasses.

In this corpus, we present nine types of entity labels, including material, regulation method, product, faradaic efficiency, cell setup, electrolyte, synthesis method, current density, and voltage. In addition, we provide a more detailed label subclass in some entity labels, such as material, regulation method and product. The description of label category is shown in Fig. 4, as well as the subclass of material, regulation method and product. For instance, when an annotator located one material that is described as electrocatalyst, he/she needs to specify what kind of this material is, such as Cu, Cu/C, CuO_*x*_, etc.

### Construction of extended corpus

As the manual annotation process is laborious, a lower quality corpus, also known as a *silver standard corpus* (SSC)^25^, was constructed using automated techniques. In this paper, we generate an extended corpus according to the construction standard of the *silver standard corpus* (SSC). The main types of entities involved in the CO_2_ electrocatalytic reduction process include materials, products, regulation methods and the corresponding Faraday efficiencies. The other physical information including cell setup, catalyst synthesis methods, current density and faradaic efficiency voltage are additional information about the CO_2_ electrocatalytic reduction process and have less annotation information, so we did not extract these information in the automatically annotated extended dataset. A schematic representation of the procedure is shown in the bottom panel in Fig. 1. In the sections below, we provide a brief overview of the methods used for each step of the Entity extraction.

#### Coarse-grained entity recognition

To identify and extract coarse-grained category entities from the full text of the literature, we implemented a bidirectional short-term memory neural network with a conditional random field layer on top of it (BiLSTM-CRF)^26,27^, which is able to recognize the semantic information of a word based on both the word itself and its context. SciBERT module^28^ is a scientific domain-oriented variant of BERT^29^, which remains the original architecture of BERT and pre-trained on scientific corpora. In such a manner, domain knowledge would be consolidated into SciBERT and therefore improves its performance on downstream tasks. First, each word token was transformed into a digitized SciBERT embedding vector. A bi-directional long-short-term memory neural network with a conditional random field top layer (BiLSTM-CRF) was used to determine the corresponding entity class labels. The annotated dataset was split into training, validation, and test sets with a paper-wise ratio of 8:1:1 to train the aforementioned neural network.

When assuming that the automated tools have an acceptable performance, the combination of multiple systems can generate labels with an acceptable quality. Considering that some material and product entities are usually described in terms of chemical formula and faradaic efficiency entity is often described in the form of numerical “value unit”, we proposed a rule-based approach to assist the model in its identification^30^. Typically, the creation of an extended corpus required corpus harmonization to merge multiple predictions. Here we consider the simplest case, applying voting schemes^25^ and various reference boundary coordination strategies (for example, accurate, nested, continuous similarity metrics for reference alignments^25^) for the final decision.

#### Fine-grained entity classification

Fine-grained entity categories divide entities in a more granular way. In order to identify and classify entities obtained from the previous task, we implemented a classification algorithm combining dictionary and maximum entropy model. The dictionary-based recognizer used a word list established on the expert-annotated data^31^. The maximum entropy model was used to extract features from the data that cannot be matched by the dictionary. The features of each entity were obtained from its word embedding vectors, context vectors, word cluster clustering information and coarse-grained entity category information through a simple mapping function. Sentences were tokenized using ChemDataExtractor’s ChemWordTokenizer^20^ in order to obtain word embedding vectors. The context vector of each word was obtained through mask training of the SciBERT model mentioned above.

Our system utilised features derived from Brown clustering^32^, which is a form of hierarchical clustering of words based on the contexts in which words occur. This has been proved to improve the performance of part-of-speech tagging and named entity recognition in various domains^33–37^. Clustering was performed on the full text and titles of 2123 material articles published by ACS, RSC and Springer. This collection contained about 20 million words out of about 700,000 sentences, with tokenization from ChemDataExtractor’s ChemWordTokenizer. Liang C++ implementation^38^ was used to perform clustering and generate 1,500 clusters containing 372,799 unique words. This clustering information was also used as the classification feature of entities for model training.

#### Calibration of the extended corpus

First we automatically revised the annotation results for the extended corpus to cross check the mention boundaries, trim whitespace characters, and ensure their technical consistence with the annotation rules. We then selected a 50% random sample from the entire dataset to be manually proofread by the main annotation team of the Golden Corpus. For potentially inconsistent cases where a given chemical name was annotated in automatic labelling as one entity class and in manual annotation as another entity class, we relied primarily on the annotations of the main annotator team because these curators had a higher degree of experience in this task and they did provide active feedback for the refinement of the annotation. After one round of a rather rough proofreading process, this corpus contained only the crude annotations. By doing this we intend to encourage follow-up researchers to explore their own downstream NER tasks, such as cross comparison, mention alignment and consensus annotations strategies. A total of 145,179 automatic annotations were generated for these 8184 paragraphs. On average, the number of entity mentions per abstract was of 17.74, almost four times when compared to the benchmark corpus. A possible reason for this was that the automatic model identified eligible entities, but the context of the entities mentioned in the text was not relevant to the CO_2_ electrocatalytic reduction process. However, it was useful to examine more difficult or easier cases and to detect potential annotation errors when examining consensus predictions generated by multiple systems.

## Data Records

The two types of datasets presented in this paper are available at Science Data Bank (ScienceDB), which is a public, general-purpose data repository aiming to provide data services for researchers, research projects/teams, journals, institutions, universities, etc.

The benchmark corpus is publicly available at 10.57760/sciencedb.07106^39^. The extended corpus is publicly available at 10.57760/sciencedb.07139^40^. The two Standard Corpus are provided as a file in CSV format, and the details of them are shown in Table 1.

**Table 1:** Summary of the two corpus.

Corpus Type	Benchmark Corpus	Extended Corpus
Entity Type		
Material	769	36651
Regulation method	769	66806
Product (including the second and third product)	1008	27045
Faradaic efficiency (including the Faradaic efficiency of second and third product)	903	14677
Cell setup	402	—
Electrolyte	447	—
Synthesis method	843	—
Current density	296	—
Voltage	649	—
Total	6,086	145,179

Metadata contained in the dataset for an article include: article DOI, the year of publication, and the title. Each record metadata includes: entity extracted from the paper, label of the entity, and the sentence where the entity is located. Expanded details for the format of the dataset are given in Table 2.

**Table 2:** Metadata of the corpus.

Data Description	Data Key Label	Data Type
DOI of the original paper	doi	string
Public year of the original paper	public_year	int
Title of the original paper	title	string
Entity extracted from the paper	entity	string
Label of the entity	entity_label	string
Sentence where the entity is located	context	string

## Technical Validation

### Extraction accuracy

To ensure high accuracy of the dataset, we only included data from the CO_2_ electrocatalytic reduction process obtained at the final filtering step of the pipeline. This strategy reduced potential errors in the dataset that may have been caused by combination-parsing failure, incomplete extraction, or incomplete information provided by the text. We applied the extraction line to 200 randomly selected documents in the material field, 150 of which were relevant to electrocatalytic reduction of CO_2_ processes over copper-based catalysts, giving an extraction rate of approximately 75%. Although these excluded documents are also relevant to the topic of our concern, they are primarily concerned with theoretical calculations, mechanism investigations, and experimental studies in organic solutions, all of which are beyond our consideration.

To demonstrate the practicality of our annotated corpus, we explored two machine learning methods for extracting actions and entities: a maximum entropy model and several neural network tagging models. We used standard precision, recall and F1 indicators to evaluate and compare performance. In the maximum entropy model^41^, we used two types of features based on the current word and context words within a window of size 2: the part-of-speech feature generated by GENIA part-of-speech Tagger^42^, which is specially adjusted for biomedical texts, and the Lexical features, including unigrams, bigrams as well as their lemmas and synonyms from WordNet^43^. Neural network annotators included the most advanced bidirectional LSTM with conditional random field (CRF) layer^27,44,45^, bidirectional recurrent neural network Bi-GRU^46^ and BERT model with conditional random field (CRF). Table 3 shows the experimental comparison results. We found that the BERT-BiLSTM-CRF model consistently outperformed other methods, achieving an overall F1 score of 81.95 at identifying four coarse-grained category entities.

**Table 3:** Compare the F1 scores of entity recognition in various models.

Entity(freq. in test set)	MaxEnt	BiLSTM-CRF	BiGRU-CRF	BERT-CRF	BERT-BiLSTM-CRF
MATERIAL(92)	43.37	49.56	50.40	57.58	**57.96**
METHOD(97)	37.97	46.35	47.88	56.45	**57.41**
PRODUCT(94)	68.25	81.88	82.16	89.97	**90.86**
FARADAIC EFFICIENCY(62)	83.68	87.56	87.98	92.12	**92.68**
Macro-avg F1	49.23	64.44	65.58	69.47	**70.12**
Micro-avg F1	68.03	78.03	80.69	81.56	**81.95**

In order to demonstrate the utility of the multi-task entity extraction, we conducted ablation experiments on the maximum entropy classification model to verify that the new features introduced are effective in improving the results^47,48^. Table 4 shows the precision, recall and F1 score of the maximum entropy classification model using various features. Parts of speech features alone are the most effective in capturing entity words. This is largely due to entity words appearing as verbs or nouns in the majority of the sentences. Cluster features are less effective in classifying method entities, because they usually have long spans and do not share similar semantic features. Our empirical results on using common machine learning algorithms such as maximum entropy model and neural network models show plenty of room for improvement when compared with the estimated domain experts’ performance, and suggest that our corpus could serve as a benchmark for evaluating material specific natural language processing research. We leave further investigation for future work, and hope the release of our dataset can help draw more attention to NLP research on instructional languages.

**Table 4:** Precision, Recall and F1 (micro-average) of the maximum entropy model for fine-grained entity classification, as each feature is added.

Classification Model	Entity Type		
Features	METHOD	MATERIAL	PRODUCT
Words	67.71	72.56	85.76
+Context(Machine-learning model)	68.93	74.52	87.49
+Entity Type(Coarse-grained)	69.81	75.73	88.16
**+Brown clusters**	**69.84**	**77.04**	**89.07**

### Dataset mining

In order to illustrate the current status and future trends of Cu-based electrocatalysts in the field of CO_2_ electroreduction, the nine types of entities in this dataset were visualized and analyzed.

**Fig. 5:**
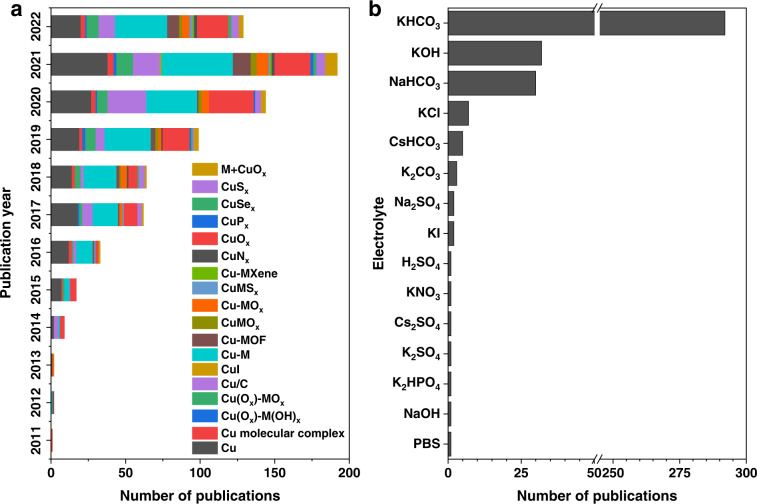
(**a**) Stacked frequencies of Cu-based electrocatalysts for CO_2_ reduction in the last 12 years. (**b**) Frequencies of different electrolytes used in CO_2_ electroreduction.

**Fig. 6:**
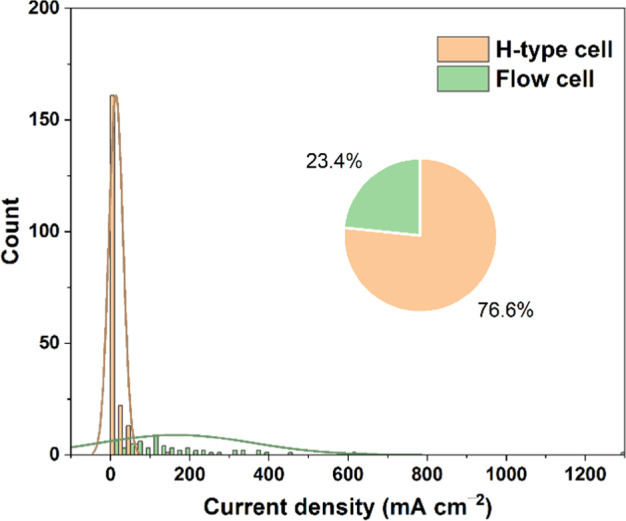
The statistical distribution of the count of current densities measured by different cell setups. Inset: The percentage of different cell setups applied in CO_2_ electroreduction.

First, we presented the overall development course of Cu-based electrocatalysts for CO_2_ reduction in the last 12 years (Fig. 5a). The number of publications on Cu-based electrocatalysts has gradually grown from several articles in 2011 to about 200 articles in 2021. The catalysts that researchers are most interested in focus on Cu, Cu-M, and CuO_*x*_, and various composite catalysts such as Cu/C, Cu(O_*x*_)-MO_*x*_, and M + CuO_*x*_ are given increasing attention nowadays. Apart from catalysts, the test condition of CO_2_ electroreduction should also be considered due to its important role in performance. In terms of electrolytes, the KHCO_3_ electrolyte is most commonly used in CO_2_ electroreduction, with KOH, and NaHCO_3_ following (Fig. 5b). Furthermore, the type of cell setup is another important test condition. As shown in Fig. 6, the statistical distribution of the count of current densities measured by different cell setups is presented. The current density of the H-type cell is largely concentrated in the values less than 20 mA cm^−2^, while that of the flow cell exhibits an average value of close to 200 mA cm^−2^, revealing the dependence of performance on cell setup.

**Fig. 7:**
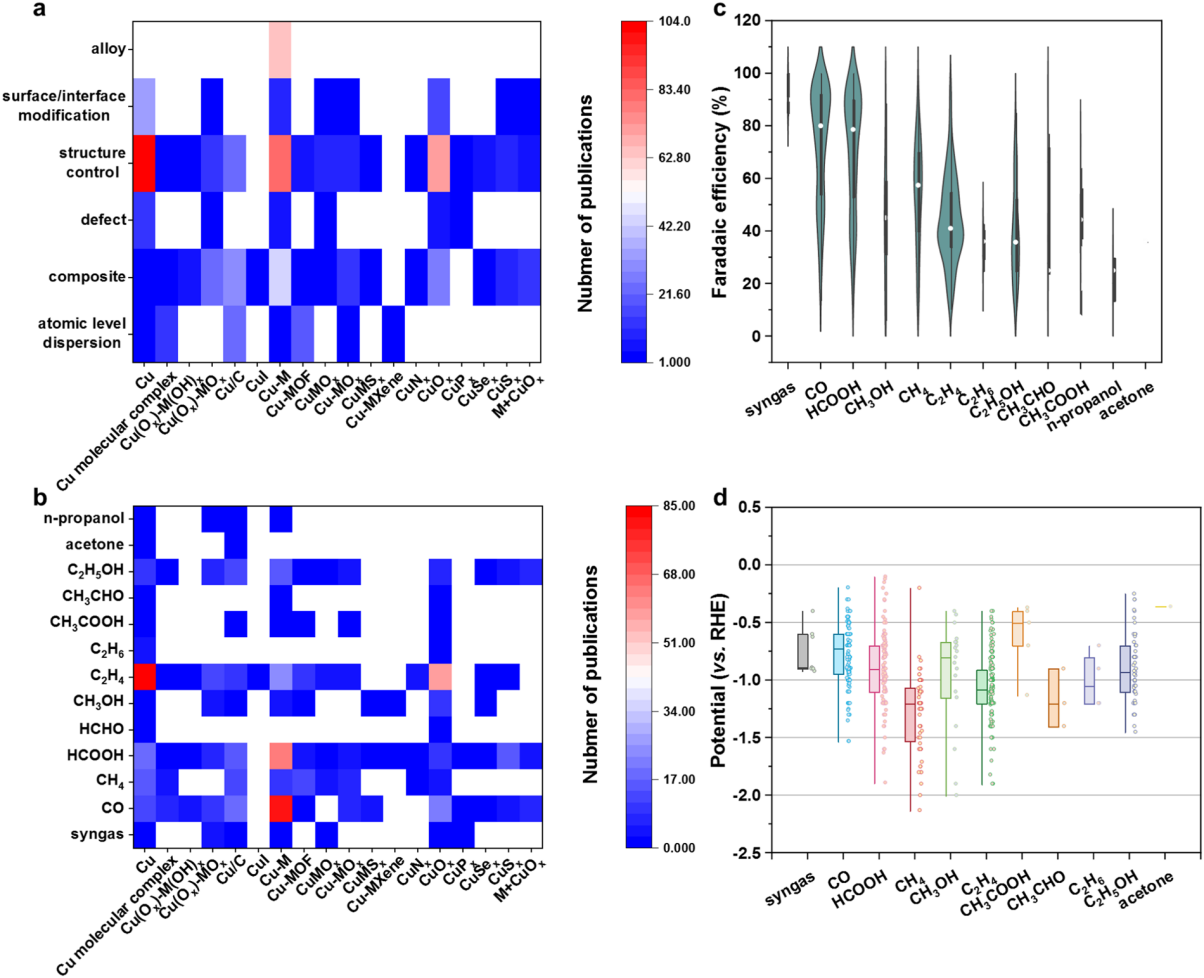
(**a**) Heatmap depicting the number of publications of Cu-based electrocatalysts with different regulation methods. (**b**) Heatmap depicting the number of publications of Cu-based electrocatalysts with various products. (**c**) Violin plots of Faradaic efficiency as a function of product. (**d**) Box plots of the potential of CO_2_ reduction as a function of product. The points alongside the boxes present the distribution of experimental results in the dataset.

In addition to catalysts and test conditions, we also analyzed the regulation method and performance of catalysts. Figure 7a shows a heatmap depicting the number of publications of Cu-based electrocatalysts with different regulation methods. The structure control approach exhibits widespread use to modify the surface morphology, structure, and crystal phase of catalysts. Other approaches deliver a high degree of correlation with the type of catalysts. For example, only the binary Cu-M systems contain the alloy form of catalysts, surface/interface modification is mainly applied in Cu surfaces, and the atomic level dispersion of Cu atoms mostly takes place in Cu molecular complexes, Cu/C, and Cu-MOF. Figure 7b shows a heatmap presenting the relationships between materials and products for CO_2_ reduction. It can be seen that Cu and CuO_*x*_ show a clear tendency to produce C_2_H_4_ and the Cu-M catalysts tend to produce C_1_ products such as CO and HCOOH. The blank area of this heatmap also presents some potential research directions for researchers. As shown in Fig. 7c, we drew violin plots of Faradaic efficiency as a function of product to illustrate the associations between products and corresponding Faradaic efficiencies in the scientific literature. The Faradaic efficiency of C_1_ products is statistically higher than C_2+_ products. Specifically, most of the articles reporting CO and HCOOH as the main products realize a Faradaic efficiency of more than 80%, while the articles related to C_2_H_4_ and C_2_H_5_OH only report a Faradaic efficiency of about 40%. These results demote the difficulty of C-C coupling to product C_2+_ products. Furthermore, we also analyzed the correlations between the potential of CO_2_ reduction and product (Fig. 7d). C_1_ products are commonly produced at a lower overpotential than C_2+_ products. This result experimentally implies that a higher reaction energy barrier is needed to generate C_2+_ products. An interesting finding needed to pay attention is that CH_3_COOH shows the lowest overpotential, and this result needs further study.

**Fig. 8:**
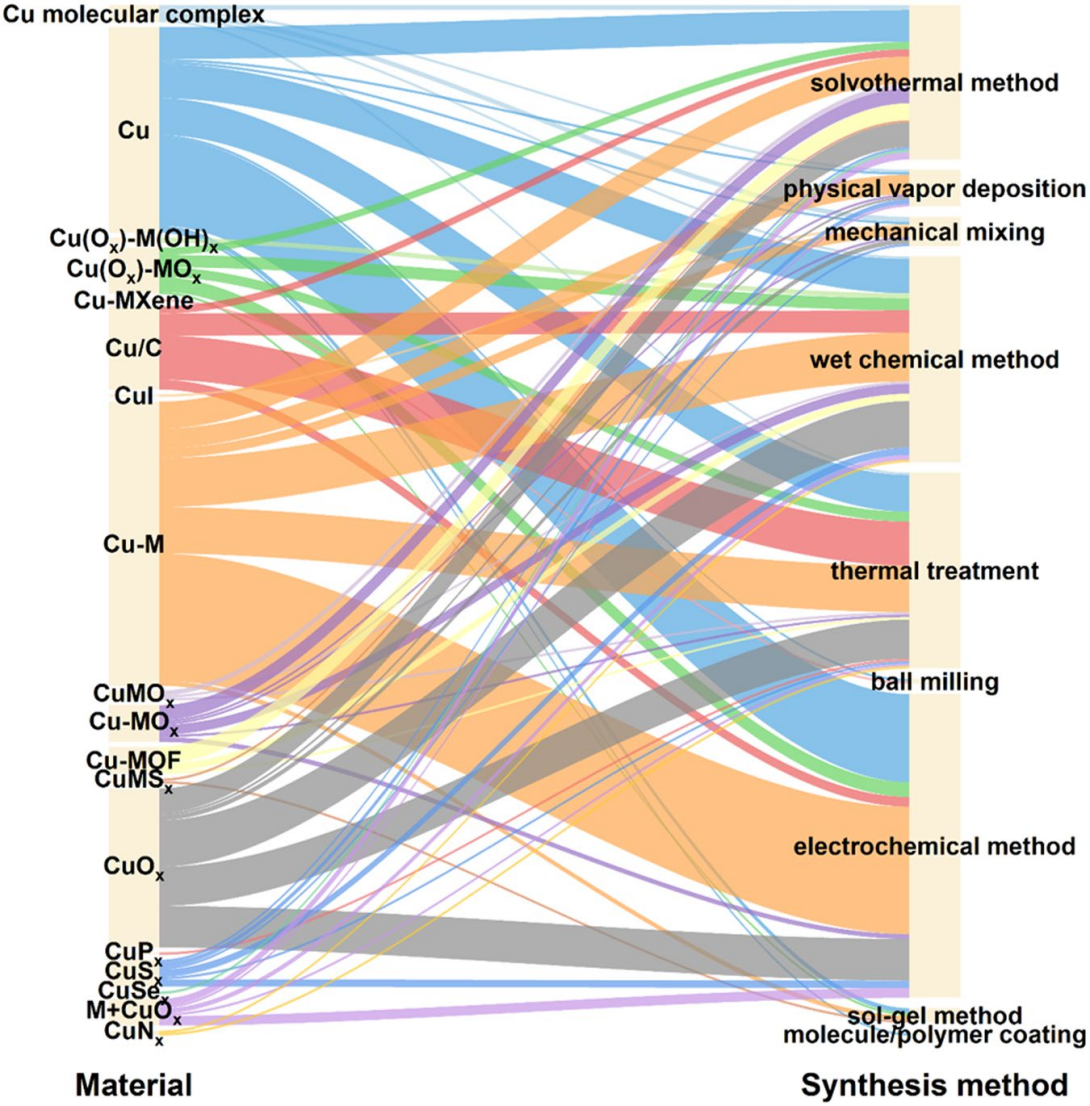
Alluvial plot showing the relationships between catalysts and synthesis methods.

Finally, we presented the relationships between catalysts and synthesis methods based on the data from the benchmark corpus. We first labeled the synthesis methods of Cu-based electrocatalysts from the full text of articles and then divided them into eight categories including balling milling, wet chemical method, electrochemical method, solvothermal method, thermal treatment, sol-gel method, mechanical mixing, physical vapor deposition, and molecular/polymer coating. As shown in Fig. 8, the wet chemical method, electrochemical method, solvothermal method, and thermal treatment are the most commonly used approaches to prepare Cu-based electrocatalysts. Some relationships between catalysts and synthesis methods can also be found in Fig. 8. For instance, the electrochemical method is the most popular approach to preparing Cu and Cu-M catalysts, Cu/C is mainly prepared by thermal treatment, and physical vapor deposition is mainly used to obtain biphase Cu-M catalysts. These results can provide an intuitional guideline for the preparation of Cu-based electrocatalysts.

## Data Availability

The scripts utilized to parse articles and extract entities are home-written codes which are publicly available at the github repository https://github.com/kg4sci/electrocatalytic_db. The underlying machine-learning libraries used in this project are all open-source: ChemDataExtractor (chemdataextractor.org)^20^, gensim (radimrehurek.com)^49^, PyMuPDF(https://github.com/pymupdf/PyMuPDFPyMuPDF), Pytorch (www.pytorch.org) and scikit-learn (scikit-learn.org)^50^.
